# Curcumin Alleviates oxLDL Induced MMP-9 and EMMPRIN Expression through the Inhibition of NF-κB and MAPK Pathways in Macrophages

**DOI:** 10.3389/fphar.2017.00062

**Published:** 2017-02-14

**Authors:** Jiatian Cao, Bozhi Ye, Lu Lin, Lei Tian, Hongbo Yang, Changqian Wang, Weijian Huang, Zhouqing Huang

**Affiliations:** ^1^Division of Cardiology, Shanghai Institute of Cardiovascular Diseases, Zhongshan Hospital, Fudan UniversityShanghai, China; ^2^Division of Cardiology, School of Medicine, Shanghai Ninth Hospital, Shanghai Jiao Tong UniversityShanghai, China; ^3^Division of Cardiology, The Key Lab of Cardiovascular Disease of Wenzhou, The First Affiliated Hospital of WenZhou Medical UniversityWenZhou, China

**Keywords:** curcumin, EMMPRIN, MMP-9, oxLDL, macrophage, atherosclerosis

## Abstract

Rupture of vulnerable atherosclerotic plaques is the leading cause of acute myocardial infarction (AMI) and unstable angina pectoris (UA). However, it still lacks an effective therapy to stabilize the vulnerable atherosclerotic plaques. Numerous reports have shown that upregulation of MMP-9 (matrix metalloproteinase-9) and EMMPRIN (extracellular matrix metalloproteinase inducer) in macrophages is involved in the progression and development of vulnerable plaques. Here we evaluated the impact of curcumin on the expression of MMP-9 and EMMPRIN in macrophages. Macrophages were pretreated with curcumin or specific inhibitors (p38 MAPK inhibitor, NF-κB p65 inhibitor) for 1 h, then cells were cultured with oxLDL for indicated time. Real-time PCR and Western blot analysis were used to evaluate the expression of mRNA and proteins. Translocation of NF-κB p65 was detected by using laser confocal microscopy. Here we showed that curcumin attenuated the MMP-9 and EMMPRIN expression in oxLDL stimulated macrophages. Further studies revealed that curcumin inhibited oxLDL induced NF-κB activation and p38 MAPK phosphorylation. These findings illustrated that curcumin can inhibit the expression of EMMPRIN and MMP-9 in oxLDL stimulated macrophages through down regulation of NF-κB and p38 MAPK signaling pathways, which might be the molecular mechanism for the anti-atherosclerotic effect of curcumin.

## Introduction

Atherosclerosis is defined as a chronic inflammatory disease in the arterial wall, and is closely correlative with accumulation of macrophages, dysfunction of endothelial cells, and activation of immune cells (Fuster and Kovacic, 2014; Zeller and Srivastava, 2014; Gimbrone and Garcia-Cardena, 2016). Monocytes recruitment and their differentiation into macrophages in the subendothelial space, macrophages take up lipids from lipoproteins and transforming into foam cells, which further enhances inflammation and atherogenesis (Tabas and Bornfeldt, 2016). Among the inducer of inflammations, Oxidized low-density lipoprotein (oxLDL) is recognized as a key activator of inflammation and a critical cardiovascular risk (Di Pietro et al., 2016). It is proved that monocyte-derived macrophages play a critical role in the atherosclerosis initiation and progression. Emerging data have demonstrated that local inflammation induced by oxLDL is the major cause of atherosclerotic plaque fracture, which leads to stroke and myocardial infarction (Libby and Aikawa, 2002; Belovici and Pandele, 2008). Since the first historical description of plaque rupture in 1,844, several key mechanisms about plaque vulnerability have been detected. Increased evidence from genetic studies and emerging clinical research suggest that MMP-9 (matrix metalloproteinase-9) is one of the most risk factors for inducing plaque rupture (Fuster and Kovacic, 2014; Kobayashi et al., 2016). MMP-9 is a member of the MMPs, which are enzymes that can degrade ECM (extracellular matrix). EMMPRIN (extracellular matrix metalloproteinase inducer), also termed CD147, is a 58-kDa cell surface glycoprotein described first in tumor cells. It has been reported to be secreted by macrophages in response to oxLDL stimulation and implicated in plaque rupture (Moreau et al., 1999; Chen et al., 2011). Monocyte-derived macrophages in atherosclerotic plaques secrete MMP-9, which contributes to plaque rupture (Cipollone et al., 2003). Over-expression of MMP-9 and EMMPRIN in monocytes/macrophages result in plaque progression and destabilization. In humans, plasma concentrations of MMPs were significantly increased in patients with acute coronary syndromes (ACS) or coronary artery disease (CAD) compared with those in healthy controls (Blankenberg et al., 2003; Fitzsimmons et al., 2007; Bencsik et al., 2015). Although recent advances have been made in PCI (percutaneous coronary intervention) and drug therapies, identifying a new plaque vulnerability therapy is still a challenging work in progress. Here, we speculate that EMMPRIN and MMP-9 would be critical therapeutic targets, suppressing the expression of them can inhibit plaque rupture or retard atherosclerosis.

Curcumin is a safe and effective natural polyphenolic compound extract from curcuma longa and turmeric and found to be very valuable in anti-cancer, anti-oxidant, and anti-inflammatory processes (Bimonte et al., 2016; Kunnumakkara et al., 2016). Studies strongly support that curcumin is of potential therapeutic value in atherosclerosis and CAD (Sikora et al., 2010; Chen et al., 2015). Moreover, curcumin suppresses oxLDL stimulated CD36 expression via p38 MAPK pathway and prevents the migration of human aortic smooth muscle cells (HASMCs) by reducing the expression of MMP-9 (Yu and Lin, 2010). In our previous study, we found curcumin decreased expression of MMP-9, MMP-13 in PMA induced human monocyte cells (Cao et al., 2014). However, the mechanisms of curcumin's effects on EMMPRIN and MMP-9 expression in macrophages have not been elucidated. In this study, we determined the regulation of MMP-9 and EMMPRIN expression by curcumin in oxLDL stimulated macrophages and identified its molecular mechanisms.

## Materials and methods

### Cell culture and methods

To evaluate the effects of curcumin on uptake of oxidized low density lipoprotein (oxLDL) in PMA induced macrophage, human monocyte cell line THP-1 (ATCC, Rockville, MD) were seeded on six-well plates at a density of 10^6^ cells per well in RPMI 1640 medium containing 10% heat-inactivated Fetal calf serum (Gibco, Grand Island, NY, USA). To induce the differentiation of THP-1, cells were treating with 100 nM phorbol 12-myristate 13-acetate (PMA, Calbiochem, San Diego, CA) for 48 h (Tsuchiya et al., 1982). To study dose-dependent effects of curcumin, the PMA induced macrophages were treated with curcumin (0 to 50 μM, Sigma, USA), 10 μM SB203580 (p38 MAPK inhibitor, Sigma, USA), or 20 μM BAY-11-7082 (NF-κB p65 inhibitor, Sigma, USA) for 1 h, and then stimulated with oxLDL (50 ug/L, Oxidized Human LDL, Peking Union-Biololgy Co. Ltd.) at the indicated time. To study time-dependent effects of curcumin, macrophages were pretreated with vehicle or curcumin (25 μM) for 1 h, followed by oxLDL (50 ug/L) for 1, 3, 6, or 12 h, then the cells were harvest for further analysis.

### RNA isolation, cDNA synthesis, and real-time PCR

Real-time PCR was performed to determine gene expression of EMMPRIN, MMP-9. Total RNA was isolated from cells using Trizol reagent (Invitrogen), cDNA was synthesized using Reverse Transcription Kit (Takara) according to the manufacturer's instructions. Quantification real-time PCR were performed by SYBR Premix Ex Taq Kit (TaKaRa Code DRR041) as previously described (Meng et al., 2012). The primer sequences of PCR are listed in Table 1. GAPDH was used for normalization.

**Table 1:** **Real time-pCR primers of MMP-9 and EMMPRIN**.

**Target genes**	**Forward (5′–3′)**	**Reverse (5′–3′)**
EMMPRIN	TTGGAGGTTGTAGGACCGGCGA	TGGGACCCTGCCCTTCAAACCA
MMP-9	TGACGCCGCTCACCTTCACT	CGCGCCATCTGCGTTTCCAA
GAPDH	CCGCATCTTCTTTTGCGTCGCC	TCTCAGCCTTGACGGTGCCA

### Preparation of cell lysates and nuclear fraction, and immunoblotting

Cells were lysed with 100 mM phenylmethanesulfonyl fluoride, and the protein extracts were denatured and loaded onto a 10% SDS-PAGE gel. To measure IκB-α phosphorylation and nuclear p65 expression levels, NE-PER nuclear and cytoplasmic extraction reagents were used to isolated protein from the cytoplasm or nuclear. Then, western blot analysis were performed as we reported previously (Huang et al., 2012; Cao et al., 2014). Briefly, membranes were first incubated with primary antibodies for Lamin B (Sigma-Aldrich, St. Louis, MO), EMMPRIN (Life Technologies, USA), MMP-9, p-p38, p38, p-ERK, ERK, p-JNK, JNK, p-IκB-α, p65, or β-actin (Cell Signaling Technology, Boston, MA) then incubated with far-red fluorescent secondary antibodies (Life technologies, USA). All signals were conducted by the Odyssey imaging system (Li-cor, USA).

### Gelatin zymography

Gelatin zymography were performed as described in our previous study (Cao et al., 2014). PMA induced macrophages were seeded in a six-well plate at the density of 3 × 10^5^ cells per well. To study dose-dependent effects of curcumin, the PMA induced macrophages were treated with curcumin (0 to 50 μM), 10 μM SB203580 (p38 MAPK inhibitor, Sigma, USA), or 20 μM BAY-11-7082 (NF-κB p65 inhibitor, Sigma, USA) for 1 h, and then stimulated with oxLDL (50 ug/L) for the indicated time. To study time-dependent effects of curcumin, macrophages were pretreated with vehicle or curcumin (25 μM) for 1 h, followed by oxLDL (50 ug/L) for 1, 3, 6, or 12 h, then the cells were harvest for further analysis. The culture supernatants were collected, 10 μl aliquots of the culture supernatant were loaded onto a 10% polyacrylamide gel containing 1 mg/ml gelatin. After electrophoresis, gels were washed twice with 2.5% Triton X-100 (37°C, 15 min) and then gels were incubated at 37°C for 11 h in developing buffer containing 10 mM Tris Base, 40 mM Tris–HCl, 200 mM NaCl, 10 mM CaCl_2_, 0.02% Brij. Gels were subsequently stained with 0.5% (w/v) Coomassie Blue R-250 for 2 h followed by destaining with a solution containing 50% methanol, 10% glacial acetic acid, 40% water. MMP-9-digested regions were visualized as light bands against a dark background. An image of each gel was detected by an Odyssey imaging system (Li-cor, USA).

### Confocal laser scanning fluorescence microscopy analyze the translocation of NF-κB

PMA induced macrophages were cultured on microcover glasses in a six-well plate, the cells were pretreated with or without 25 μM curcumin 1 h, then they were stimulated with oxLDL in the indicated time (1, 2, 3, and 6 h). The macrophages were labeled with immunofluorescence using cellular NF-κB translocation kit (Beyotime Biotech) as described in our previous study (Huang et al., 2012). Briefly, at timed intervals, cells were fixed and permeabilized, after staining with NF-κB p65 antibody, cells were incubated with a rabbit IgG antibody conjugated with Cy3. The Nucleus were stained by DAPI, after the cells were incubated with it for 5 min. The activation of NF-κB p65 was visualized by using confocal microscopy (FluoViewTM FV1000, Olympus).

### Statistical analysis

Multiple group comparisons were performed using oneway ANOVA. *P* < 0.05 was considered statistically significant. All experiments were performed at least three times.

## Results

### Curcumin inhibited the expression of MMP-9 and EMMPRIN in oxLDL-stimulated macrophages

**Figure 1:**
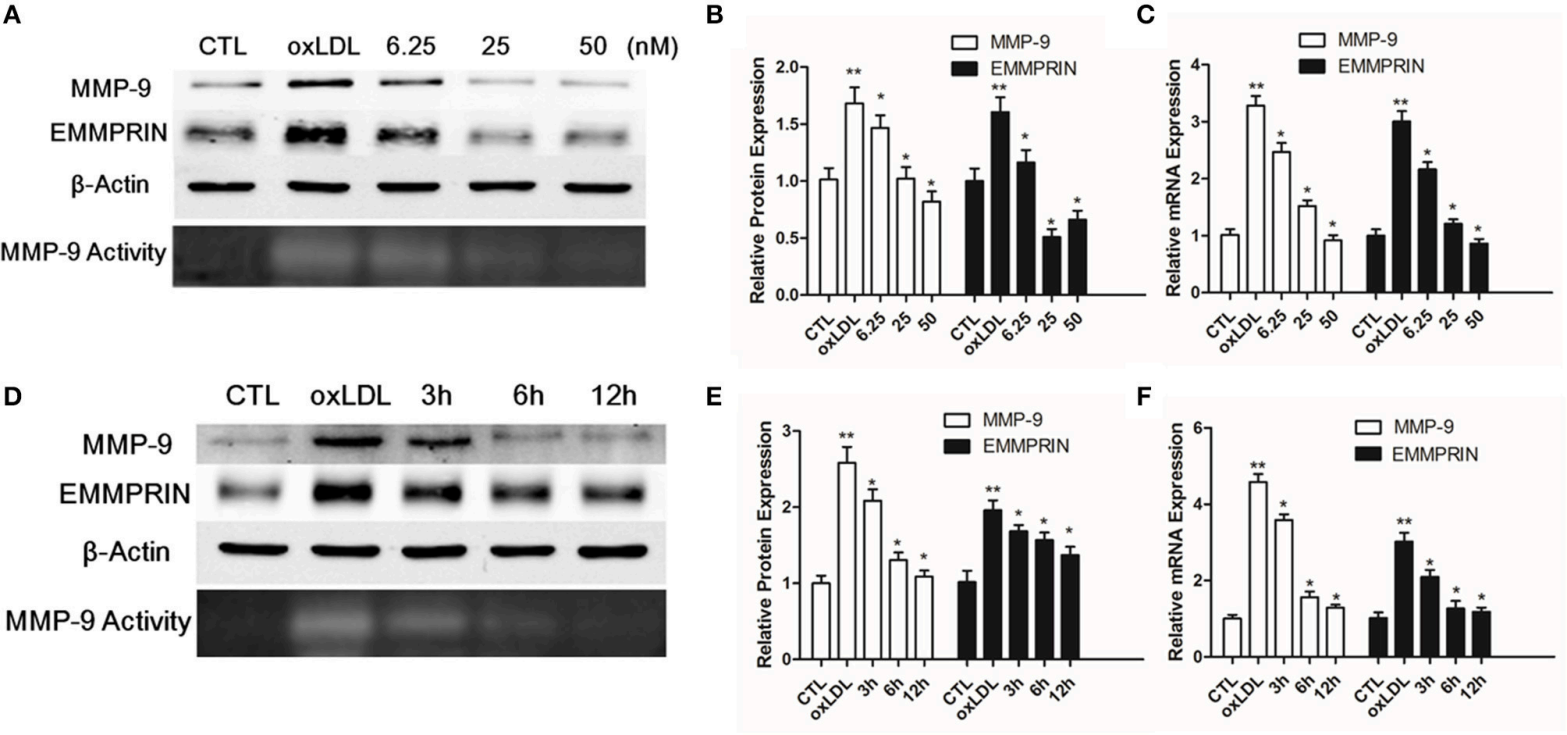
**Curcumin inhibits MMP-9 and EMMPRIN in oxLDL-stimulated macrophages**. **(A–D)** Curcumin suppresses EMMPRIN and MMP-9 expression in oxLDL-stimulated macrophages at dose-dependent manner (6.25, 25, and 50 μM). **(E,F)** Curcumin reduced MMP-9 and EMMPRIN expression at time-dependent manner (3, 6, and 12 h). Real-time PCR and Western blot were used to measure the mRNA and protein level. ^*^P < 0.05 vs. oxLDL group, ^**^P < 0.01 vs. CTL group (Control group). CTL group suggest the cells incubated in a medium with DMSO. 6.25, 25, and 50 μM group suggest that indicated concentrations of curcumin were used to pretreated macrophages for 1 h, and then they were stimulated with 50 ug/L oxLDL for another 12 h. 3, 6, and 12 h indicates that macrophage were pretreated with curcumin (25 μM) for different time intervals (3, 6, and 12 h).

Elevated MMP-9 and EMMPRIN expression level was previously reported in oxLDL-stimulated macrophage. Because EMMPRIN can up-regulate the expression of MMP-9, we first need to know whether curcumin has an effect on MMP-9 expression. We pretreated THP-1 differentiated macrophages with different concentrations of curcumin (6.25–50 μM) for 1 h, then the cells were stimulated with 50 ug/L oxLDL for 12 h. Based on the western blot and real-time PCR analysis, we found that curcumin inhibited MMP-9 expression (Figures 1A–C) at a dose-dependent manner. Also, as shown in Figure 1, curcumin inhibited EMMPRIN expression. Furthermore, THP-1 differentiated macrophages were pretreated with curcumin (25 μM) for 1 h, then the expression level of MMP-9 and EMMPRIN were measured at different time points (3, 6, and 12 h) (Figures 1D–F). Curcumin significantly reduced the MMP-9 and EMMPRIN expression at a time-dependent manner. These results demonstrated that curcumin can inhibit the MMP-9 and EMMPRIN expression in oxLDL-stimulated macrophages.

### Curcumin suppressed MMP-9 and EMMPRIN expression by inhibiting activation of NF-κB

**Figure 2:**
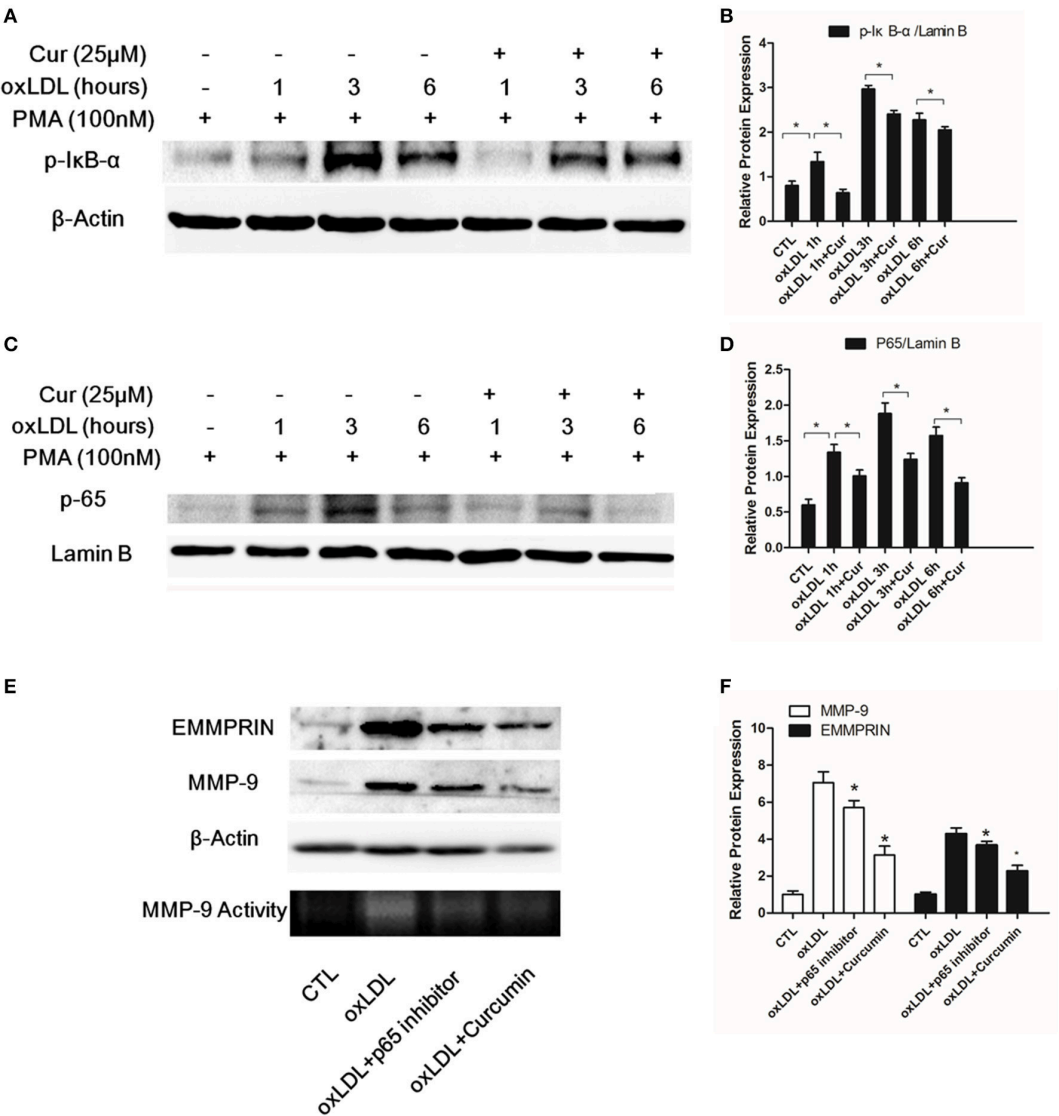
**Curcumin suppresses the phosphorylation of IκB-α (p-IκB-α) and nuclear translocation of p65 in oxLDL-stimulated macrophages**. Macrophages were pretreated with vehicle or curcumin (25 μM) for 1 h, followed by oxLDL (50 uFg/L) for 1, 3, and 6 h. The key protein levels, p-IκB-α in the cytosolic part and NF-κB p65 subunit, were assessed by Western blot analysis. **(A,B)** Effect of curcumin on phosphorylation of IκB-α in oxLDL-stimulated macrophages at different time points. **(C,D)** Effect of curcumin on p65 expression (p65 translocation) at different time points. **(E,F)** Macrophages were pretreated with vehicle, curcumin or p65-specific inhibitor (BAY-11-7082, 20 μM). p65-specific inhibitor significantly decreased MMP-9 and EMMPRIN expression. ^*^P < 0.05 when compared with the control group.

**Figure 3:**
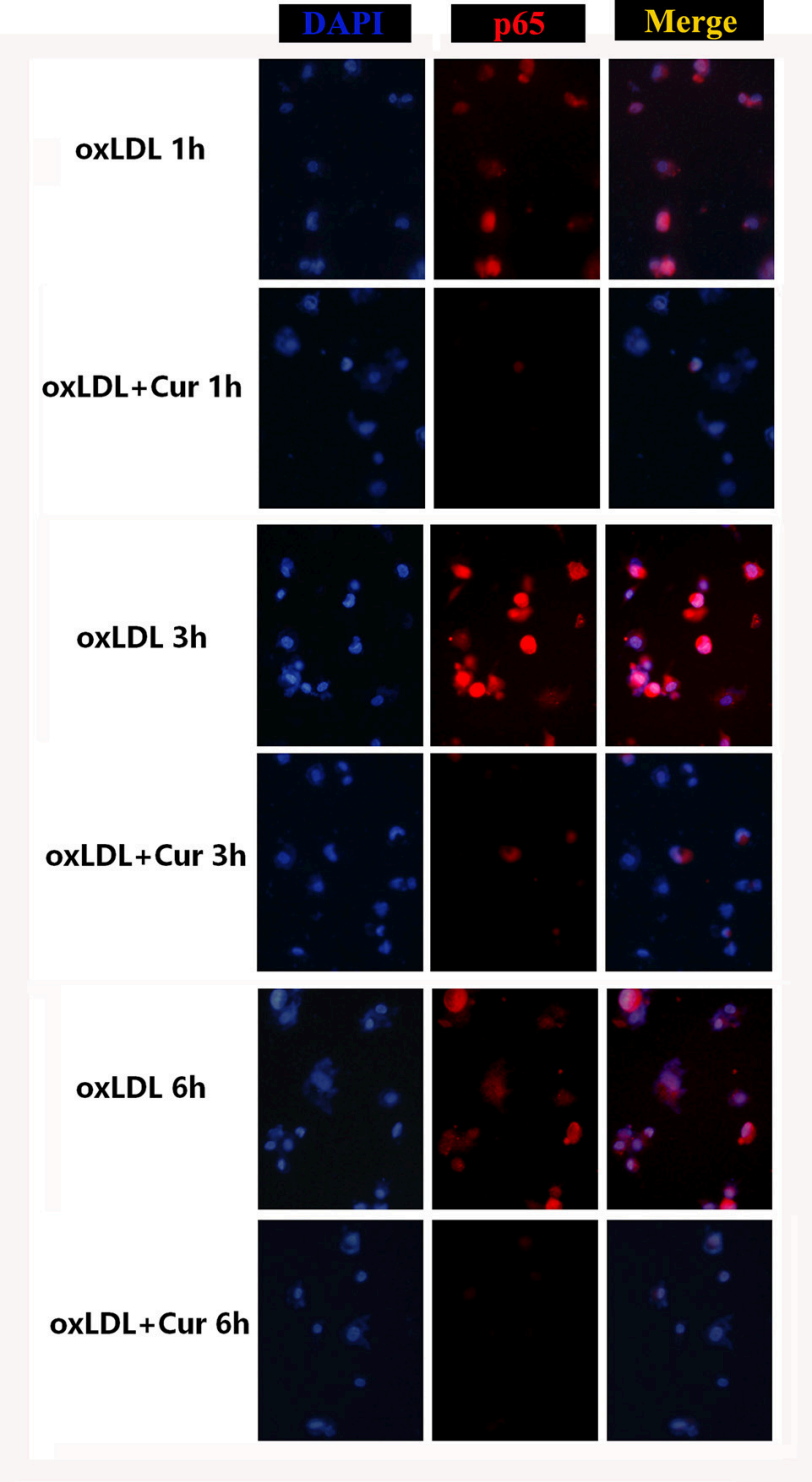
**Confocal laser scanning microscope observe the effect of curcumin on the translocation of NF-κB p65 in oxLDL-treated macrophages**. The Cy3-conjugated secondary antibody was used to analyze the localization of NF-κB p65. Cell nucleus were stained by DAPI. The fluorescence images were observed by confocal laser scanning microscope, Red marked NF-κB p65, light blue represents nuclei with DAPI. Images were merged and a purple fluorescence indicate the areas of co-localization (three independent experiments). THP-1 induced macrophages were pretreated with vehicle or curcumin (25 μM) for 1 h, and exposed to oxLDL (50 ug/L) for 1, 3, and 6 h. It was found a significant translocation of p65 to the cell nucleus after cells were stimulated with oxLDL for 3 h. In treatment groups, cells were pretreated with curcumin (25 μM) for indicated times, NF-κB p65 was retained significantly in the cytoplasm.

As a nuclear transcription factor, NF-κB plays a key role for regulating the expression of genes involved in inflammation. The NF-κB mediated pathway was involved in MMP-9 and EMMPRIN expression in previous study (Kim et al., 2009; Huang et al., 2012), we thus determined to test whether the inhibition of MMP9 and EMMPRIN expression in oxLDL-simulated macrophages by curcumin is through regulating NF-κB pathway. Nuclear protein extraction was quantified by western blot for the p65 subunit and in oxLDL (50 ug/L) stimulated macrophages, level of p65 subunit in nuclear was increased (Figures 2C,D). Meanwhile, the phosphorylation of IκB-α (p-IκB-α) in cytoplasm was elevated at indicated times (1, 3, and 6 h) (Figures 2A,B). In contrast, curcumin (25 μM) treatment blocked the nuclear translocation of p65 (Figures 2C,D) and the phosphorylation of IκB-α (Figures 2A,B). As shown in Figure 3, the same tendency was found, the stain levels of p65 diminished in curcumin-treated macrophages. In addition, we examine MMP-9 and EMMPRIN expression in oxLDL-stimulated macrophages after treated with p65-specific inhibitor (BAY-11-7082). As show in Figures 2E,F, blockade of p65 attenuated the expression of MMP-9 and EMMPRIN. In summary, our results suggested that curcumin significantly suppresses MMP-9 and EMMPRIN expression via the inhibition of NF-κB signaling pathway.

### Curcumin reduced oxLDL stimulated MMP-9 and EMMPRIN expression by inhibiting p38 MAPK phosphorylation

**Figure 4:**
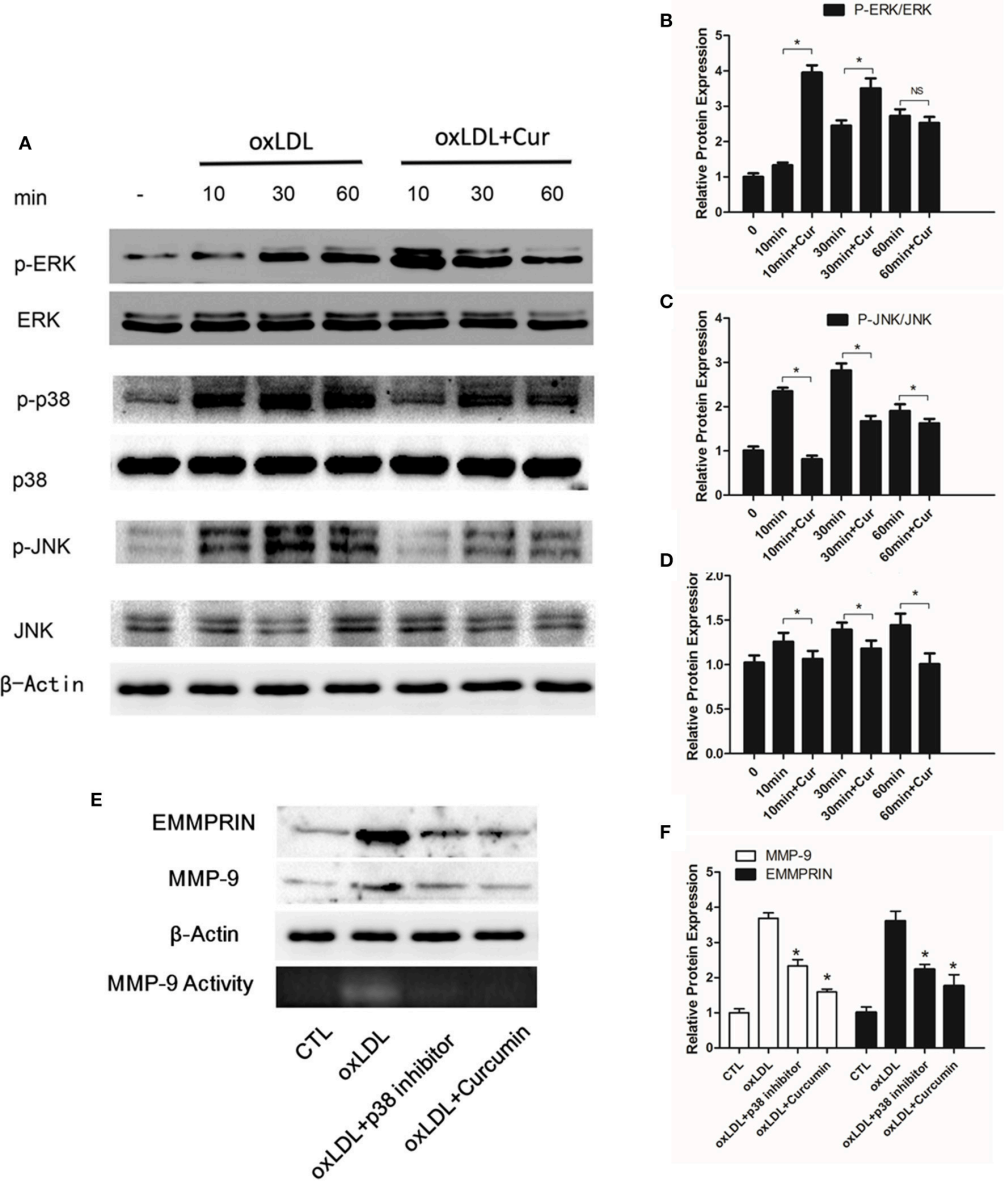
**Curcumin inhibits p38 and JNK phosphorylation. (A)** Macrophages were pretreated with vehicle or curcumin (25 μM) for 1 h, and then treated with 50 μg/ml oxLDL for 10, 20, and 60 min, and assayed by Western blot using indicated antibodies. **(B–D)** Protein quantification was carried out by densitometric analysis. Normalized proteins of JNK, p-JNK, ERK, p-ERK, p38, and p-p38 were normalized based on the internal control β-actin. **(E,F)** Macrophages were pretreated with vehicle, curcumin or specific p38 inhibitor (SB203580, 10 μM). The addition of a specific p38 inhibitor efficiently blocks oxLDL-stimulated MMP-9 and EMMPRIN expression. ^*^P < 0.05 when compared with the control group, and (NS) *P* > 0.05 when compared with the control group.

It has been previously reported oxLDL can stimulate the MAPK pathways, including ERK, p38 and JNK. To elucidate whether curcumin inhibited oxLDL-induced MMP-9 and EMMPRIN expression through regulating MAPK signaling pathways, we pretreated the THP-1 differentiated macrophages for 1 h, then cells were cultured with oxLDL for 10, 30, or 60 min. Western blot data showed that curcumin inhibited p38 MAPK phosphorylation induced by oxLDL (Figures 4A,D). To further confirm that the MMP-9 and EMMPRIN expression can be inhibited through the inhibition of p38 MAPK phosphorylation, we examined their expression in oxLDL stimulated macrophages after treated with p38-specific inhibitor (SB203580). Blockade of p38 MAPK attenuated MMP-9 and EMMPRIN expression (Figures 4E,F). In addition, curcumin also inhibit the phosphorylation of JNK (Figures 4A,C), whereas it showed no inhibitory effect on phosphorylation of ERK (Figures 4A,B). Here, the data indicated that oxLDL-induced MMP-9 and EMMPRIN expression, in THP-1 differentiated macrophages is associated with the p38 MAPK pathway and that curcumin may suppress them via this signaling pathway.

## Discussion

The treatment of ACS has been significantly improved by statins and drug-eluting stents. However, coronary events continue to be the leading cause of death over the development countries. Coronary artery plaque vulnerability and rupture is the primary cause of ACS. Accumulating evidence now indicate that overexpression of MMP-9 and EMMPRIN is critical in the development of plaque formation and rupture. Here, we found that curcumin effectively suppressed the mRNA and protein expression of both MMP-9 and EMMPRIN in oxLDL-stimulated macrophages.

The role of curcumin in foam cell formation and inflammation has been previously reported (Hasan et al., 2014). Moreover, our previous studies suggested that curcumin down-regulated MMPs expression in monocytes/macrophages transdifferentiation (Cao et al., 2014), Huang et al. also showed curcuminoids suppress matrix invasion during monocyte-macrophage differentiation (Huang et al., 2015). There is strong evidence that oxLDL plays a critical role in atherogenesis and profoundly influences the stability of atherosclerotic plaques (Zeibig et al., 2011; Pawlak et al., 2012). Monocytes/macrophages play an essential role during the various stages of atherosclerosis, including stabilization of atherosclerotic plaque, especially EMMPRIN and MMP-9 overexpression by macrophages in patient with high oxLDL (Schmidt et al., 2008; Gostiljac et al., 2011).

In the present study, our results showed that curcumin significantly reduced MMP-9 and EMMPRIN expression under the stimulation of oxLDL in macrophages, which means that curcumin could be a potential therapeutic agent for inhibiting plaque rupture or retarding atherosclerosis. oxLDL increased EMMPRIN and MMP-9 expression through NF-κB and p38 MAPK pathways and the role of elevated NF-κB and p38 MAPK activity in oxLDL induced atherosclerotic plaque rupture was previous demonstrated (Kojima et al., 2010; Yi et al., 2012), which is in agreement with other studies (Kim et al., 2001; Xie et al., 2011; Radhika and Sudhakaran, 2013). Results presented here indicated that NF-κB and p38 MAPK is constitutively active in oxLDL stimulated macrophages. NF-κB has been reported to be the most important factor regarding oxLDL induced EMMPRIN and MMP-9 activation (Singh et al., 2008; Baker et al., 2011; Park and Hong, 2016). Studies have reported that the mutation of MMPs promoters area which binds to NF-κB resulted in downregulation of MMPs in macrophages, and overexpression of IκB-α protein had the same effect (Monaco et al., 2004; Ogawa et al., 2004; Rhee et al., 2007). Therefore, NF-κB is a promising therapeutic target for ameliorating plaque formation and rupture. Our study showed that curcumin blocked NF-κB p65 nuclear translocation and inhibited the phosphorylation of IκB-α stimulated by oxLDL in macrophages, indicating that NF-κB activation was inhibited by curcumin. Our study further demonstrated that oxLDL induced EMMPRIN and MMP-9 expression can be reduced by NF-κB inhibitor. Consistent with our data, curcumin was shown to inhibit the activation of NF-κB (Kao et al., 2016; Ruiz de Porras et al., 2016), Bharat et al. has reported curcumin down-regulates the constitutive activation of NF-κB and IκBα kinase in human multiple myeloma cells (Bharti et al., 2003).

In this study, we found that oxLDL induced the phosphorylation of p38, JNK, and ERK1/2. Through inhibiting p38 and JNK phosphorylation, curcumin decreased the expression of MMP-9 and EMMPRIN in macrophages. Previous data indicated that EMMPRIN and MMPs can be regulated by p38 and JNK pathways and oxLDL triggered foam-cell formation through activation of p38 and JNK pathways (Kojima et al., 2010; Gao et al., 2013; Namgaladze et al., 2013; Liang et al., 2016). It has been reported that curcumin modulated oxLDL-induced CD36 expression via the inhibition of p38 MAPK phosphorylation in RAW 264.7 murine macrophages (Min et al., 2013). Moreover, the effects of curcumin on MAPK signaling may through inhibition of HER2 neu (Hong et al., 1999). Taken together, these findings indicated that curcumin may decrease EMMPRIN and MMP-9 expression by inhibiting NF-κB and p38 MAPK activity.

To summarize, our study demonstrated that curcumin alleviated ox-LDL induced MMP-9 and EMMPRIN expression in oxLDL stimulated macrophages, which was significantly mediated by modulating NF-κB and MAPK signaling pathways. EMMPRIN and MMP-9 represent novel targets to mitigate plaque development and diminish the burden coronary heart disease. Thus, our work opens up a new insight into the regulatory mechanism of EMMPRIN and MMP-9 in combating plaque rupture by curcumin.

## Author contributions

ZH, JC, and CW substantial contributions to the conception or design of the work. JC, BY, and ZH performed the experiments. LL, LT, HY, CW, and WH are involved in interpreting the data and provide the experimental materials. ZH and JC supervised the project and wrote the manuscript.

## Funding

This work was supported by the Chinese National Natural Science Foundation Grants (Nos. 81500382 and 81670227), Traditional Chinese Medicine Administration of Zhejiang Province (No. 2016ZA137), and Wenzhou Science & Technology Bureau (Y20150036 and Y20150035). Research Fund for the Doctoral Program of Higher Education of China (20130072110016).

### Conflict of interest statement

The authors declare that the research was conducted in the absence of any commercial or financial relationships that could be construed as a potential conflict of interest.
